# The science of story characters: a neuroimaging perspective on antagonists in narrative engagement

**DOI:** 10.3389/fnhum.2025.1569170

**Published:** 2025-05-19

**Authors:** Alejandra Obando Yar, Carmen Moret-Tatay, José Vicente Esteve Rodrigo

**Affiliations:** ^1^Doctoral School, Catholic University of Valencia San Vicente Mártir, Valencia, Spain; ^2^MEB Lab, Faculty of Psychology, Universidad Católica de Valencia San Vicente Mártir, Valencia, Spain

**Keywords:** narrative perception, character engagement, fMRI, default mode network, moral cognition, empathy

## Abstract

**Introduction:**

Understanding how humans perceive and engage with fictional characters—particularly antagonists—offers valuable insights into narrative comprehension and moral cognition. Antagonists, as morally complex figures, challenge readers’ ethical frameworks and stimulate affective and cognitive responses. This review aims to synthesize current research on the neural mechanisms involved in the perception and evaluation of antagonistic characters in narrative contexts.

**Methods:**

This systematic review analyzed findings from functional magnetic resonance imaging (fMRI) studies investigating brain activity during narrative processing, with a focus on moral and emotional evaluations of fictional antagonists. Studies were selected based on relevance, methodological rigor, and use of narrative-based stimuli. Key variables included participant demographics, types of antagonists, and narrative formats used in experimental paradigms.

**Results:**

Findings indicate that the default mode network (DMN), particularly the medial prefrontal cortex (mPFC), is consistently activated during moral and emotional evaluations of characters. Neural synchronization in areas such as the inferior frontal gyrus (IFG) and anterior cingulate cortex (ACC) suggests that character relatability significantly influences engagement and brain activity. Variations across studies point to the influence of narrative style and participant background on neural responses.

**Discussion:**

The evidence highlights the central role of the DMN in processing complex moral narratives and suggests that engagement with antagonistic characters involves both emotional resonance and ethical judgment. However, the generalizability of findings is limited by factors such as a lack of demographic diversity and inconsistent experimental designs. Future research should prioritize ecologically valid methodologies and diverse participant samples. This review emphasizes the need for interdisciplinary approaches combining neuroscience, psychology, and literary studies to deepen our understanding of narrative engagement.

## Introduction

Our cognition is built to respond to images and pictures with speed and efficiency spanning from ancient cave paintings to modern cinema. This becomes even more clear when considering stories where the audience needs to relate and engage. In such instances, characters play a fundamental role (Eekhof et al., 2023). However, beyond mere visual stimuli, humans are naturally drawn to stories. Narratives are one of the primary means through which humans communicate, understand the world, and pass down knowledge across generations (Hausknecht et al., 2021; Hecker et al., 2018).

In cinema, film, literature, and other forms of media, we are inclined to feel drawn towards certain personalities that share traits with us, whether it be through our aspirations or the struggles we have faced (Cohen, 2013). This connection can be understood through the lens of empathy. Strongly developed characters allow us to look through their person’s perspective and feel their emotions like they are our own (Cohen, 2018; Moret-Tatay et al., 2022; Niemiec and Wedding, 2013). This emotional engagement, together with well-crafted, vivid characters is what creates powerful stories. By building deeper relations with the audience, storytellers foster not only entertainment but also make people curious and make them reflect upon the scenario presented (Fernandez-Quintanilla, 2020).

While protagonists often evoke strong identification and empathy, antagonists induce moral and psychological complexity, triggering different cognitive and emotional responses in the audience. Unlike protagonist, whose narratives are usually structured to align with audience expectations, antagonists challenge moral perspectives, inducing ambivalence and cognitive conflict (Ron et al., 2022). Some villains elicit fear or disdain, while others, especially those with depth and nuance, captivate audiences despite their moral ambiguous actions (Weber et al., 2024). This variability in audience response suggest that different neural mechanisms are engaged when processing protagonists versus antagonists.

From a neuroscience perspective, narrative engagement involves a complex interplay of cognitive and emotional processes, primarily driven by neural mechanisms that support attention, memory, and social cognition. When individuals engage with a story, the brain recruits multiple regions, including the DMN—involved in self-referential thought and mental simulation (Ron et al., 2022; Yeshurun et al., 2021)—and the dorsolateral prefrontal cortex (DLPFC), which helps maintain attention and regulate emotional responses (Whitney et al., 2009). Studies using functional magnetic resonance imaging (fMRI) have shown that neural synchronization across listeners increases when they are highly immersed in a narrative, indicating shared mental representations and deeper cognitive alignment (Hasson et al., 2004). Additionally, the temporo-parietal junction (TPJ) and the mPFC are crucial for processing character intentions and emotions, allowing the audience to relate to the story on a personal level (Mar, 2011). Dopaminergic pathways linked to reward processing, particularly in the ventral striatum (STRv), reinforce engagement by making storytelling intrinsically pleasurable (Zacks et al., 2007). In essence, engagement in a narrative relies on a dynamic integration of memory, emotion, and social cognition, shaping how we understand and connect with stories on both intellectual and emotional levels.

This approach is reinforced by recent research indicates that storytelling activates brain networks associated with understanding characters’ thoughts, feelings, and intentions, suggesting a character-driven mechanism of narrative processing in the brain (Ryu and Kim, 2024). Additionally, a study by Ohad and Yeshurun (2023) found distinct patterns of neural synchronization when participants were exposed to characters with varying degrees of narrative appeal. The study showed that characters with high appeal—those with significant psychological depth and development within the story—induced greater synchronization in the DMN, reflecting a stronger cognitive and emotional engagement among participants. In contrast, characters with lower narrative appeal were associated with more dispersed synchronization, indicating reduced engagement and a more superficial perception of their role in the story. These findings underscore the importance of the DMN in processing and differentiating between different character roles within a story.

Despite its growing interest, the number of publications in the field of narrative engagement in neuroscience remains relatively limited compared to other well-established topics such as memory, attention, or motor control. While some studies have explored the neural basis of storytelling and comprehension, the field still lacks extensive empirical research and large-scale investigations. One of the key challenges is the complexity of describing and integrating the various anatomical areas involved in narrative engagement, as these processes involve a distributed network across multiple cognitive domains, including attention, emotion, and social cognition. The methodologies used to study these processes vary widely, from neuroimaging techniques such as fMRI and EEG to behavioral and psychological approaches, leading to discrepancies in findings and interpretations. Moreover, to our knowledge, no comprehensive systematic review has been conducted to consolidate the existing evidence on the neural processing of antagonists, compare methodologies, and outline a cohesive framework for understanding how the brain engages with narratives and morally complex characters. This gap highlights the need for more structured and integrative research efforts to advance the field and provide a clearer understanding of the neural mechanisms underlying narrative processing.

In light of these gaps, the present study aims to conduct a systematic review on the neural mechanisms of narrative character perception, specifically focusing on antagonists. By analysing fMRI studies, this review explores how the brain differentiates protagonist from antagonists, how moral judgments influence audience engagement, and how character complexity affects neural synchronization. In this scenario, we discuss methodological challenges, propose future research directions, and highlight the potential of naturalistic neuroimaging paradigms to bridge the gap between controlled experimental conditions and real-world storytelling experiences.

## Method

This study follows a systematic review methodology, aiming to synthesize existing neuroscientific research on how the brain differentiates antagonists from protagonists in fictional narratives. Following PRISMA (Preferred Reporting Items for Systematic Reviews and Meta-Analyses) guidelines, this review systematically identifies, evaluates, and synthesizes peer-reviewed literature on how neuroscientific mechanisms shape the perception of fictional antagonists.

### Search strategy

To identify relevant studies, a comprehensive literature search was conducted in EBSCOhost, including all its available databases, ensuring a broad and interdisciplinary approach to neuroscientific and narrative-related research. The search was performed using the following Boolean search syntax: *(“fMRI” OR “default mode network” OR DMN OR “brain activity”) AND (“protagonist” OR “antagonist” OR “villain” OR “characters” OR “fictional roles”) AND (empathy OR emotion OR “social cognition” OR “moral reasoning”) AND narratives.*

The following EBSCOhost databases were included in the search: (i) PsycINFO (for psychology and cognitive neuroscience research); (ii) MEDLINE (for biomedical and neuroimaging studies); (iii) Academic Search Complete (for multidisciplinary peer-reviewed research); (iv) Communication & Mass Media Complete (for narrative and media studies); (v) ERIC (for literature on learning, cognition, and media consumption); (vi) SocINDEX (for studies related to social cognition and moral reasoning); (vii) Film & Television Literature Index (for media and character analysis in fiction).

### Inclusion and exclusion criteria

To ensure relevance and quality, studies were included or excluded based on the following criteria:

Inclusion/exclusion criteria: The inclusion criteria focused on empirical research using fMRI or other neuroimaging techniques to examine brain activity related to narrative engagement. Studies were considered if they investigated empathy, emotional engagement, social cognition, or moral reasoning in relation to fictional characters, ensuring a direct link between neuroscience and character perception. Additionally, only peer-reviewed articles published in English were included to maintain academic rigor and accessibility.

Conversely, studies were excluded if they did not employ neuroimaging methodologies, such as purely theoretical or behavioral research, as these would not provide direct insight into the neural mechanisms underlying character perception. Studies conducted exclusively on clinical populations, such as individuals with neurological disorders, were excluded unless they directly related to narrative processing, e.g., some clinical papers used “narrative” to refer to patient histories or therapeutic storytelling without involving neural processing of narratives—those were excluded. Finally, non-peer-reviewed literature, including book chapters, dissertations, conference abstracts, and opinion pieces, was not considered to ensure the inclusion of studies with robust scientific validation. There was not limit of year publication applied.

### Data extraction and analysis

To ensure the rigor and objectivity of the systematic review, two independent reviewers screen results. In cases where there was disagreement regarding the inclusion of a study (three conflicting cases), a third independent reviewer was consulted to make the final decision. In this way, and after conducting the database search, all retrieved articles were screened for relevance based on their title and abstract, with only those meeting the inclusion criteria advancing to a full-text review. During this process, key data were systematically extracted and analyzed, including study details (authors, year, and journal), sample size and demographics (age, gender, and participant characteristics), neuroimaging methodology (fMRI parameters, task design, and regions of interest), and the narrative stimuli used (films, written texts, audiobooks, or animated media). Furthermore, the main findings related to how the DMN, and other neural mechanisms process antagonists were documented, emphasizing their theoretical contributions to empathy, moral cognition, and emotional engagement.

### Risk of bias and quality assessment

The extracted data were synthesized qualitatively, identifying common variables of interest. Following a similar system than the Newcastle–Ottawa Scale (NOS) for observational research and the Joanna Briggs Institute (JBI) Checklist for neuroimaging studies, a table was created evaluating factors such as study design rigor, sample size, and control conditions in fMRI experiments. Only studies with moderate-to-high methodological quality were included in the final synthesis.

## Results

**Figure 1:**
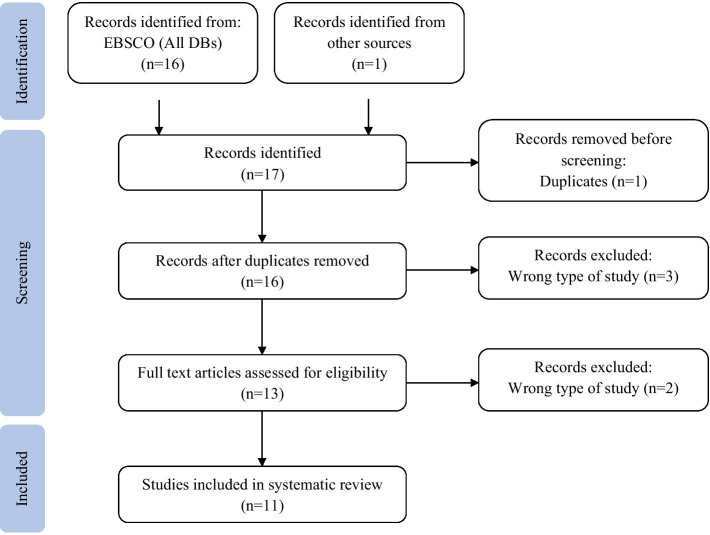
PRISMA flow diagram for systematic review: identification, screening, eligibility, and inclusion of studies in the current study.

The PRISMA flowchart illustrates the decision-making process for study selection, outlining each step from the initial database search to the final inclusion of studies. It details the number of records identified, screened, and excluded based on the inclusion and exclusion criteria. The flowchart also accounts for duplicate removal, full-text reviews, and studies assessed for methodological quality. After applying these criteria and resolving discrepancies through independent review, a total of 11 studies were included in the final synthesis. This structured approach ensures transparency and replicability in the selection process (see Figure 1).

The results from the includes studies reveal diverse neural mechanisms underlying narrative processing, with a particular focus on emotional engagement, social cognition, and moral evaluation. Table 1 highlights the methodological details and contrasts examined in each study, while Table 2 provides an overview of risk of bias evaluation.

**Table 1:** Included manuscripts in the review.

Authors	Objective	*N* (female)	Stimuli type (A, T, I)	Stimuli format (S, C, P)	Task/response	Contrast	Involved areas
Altmann et al. (2012)	Emotional valence	24 (12)	T	S	Read silently, rate valence	Negative vs. neutral valence	mPFC, IFG, TPJ, AMY
Chang et al. (2021)	Narrative integration	25 (14)	A	P	Listen, recall segments	Relevant vs. irrelevant input	DMN, HPC
Hartung et al. (2017)	Story comprehension	52 (29)	A	P	Listen, rate engagement	Differences in narrative content	mPFC, PPC, TL
Kaplan et al. (2017)	Protected values	78 (37)	T	P	Read, evaluate values	Protected vs. neutral values	mPFC, PMC, TPJ
Ohad and Yeshurun (2023)	Neural synchronization	25 (13)	A	P	Listen, rate engagement	Social vs. non-social engagement	DMN
Richardson et al. (2020)	Empathy and engagement	37 (18)	A, I	S, C	Listen, watch, self-rate empathy	High vs. low empathy scores	DMN, ACC
Ron et al. (2022)	Narrative roles	17 (7)	I	S	View faces, categorize roles	Protagonist vs. antagonist	dmPFC, PCun, TPJ, OFA
Ryu and Kim (2024)	Neural synchronization	39 (20)	I	C	Watch, empathize	Protagonist vs. antagonist	IFG, DMN, ACC
Vemuri and Surampudi (2015)	Empathy in narratives	15 (5)	I	C	Watch, empathize	Emotional vs. cognitive empathy	MDN, EMN
Wagner et al. (2016)	Social interactions	34 (27)	I	C	Watch, comprehend	Social vs. non-social interaction	dmPFC, ITC, FuG
Weber et al. (2024)	Moral norm enforcement	28 (28)	I	C	Watch, evaluate punishment, rate moral behavior and outcomes	Punished vs. rewarded immorality	mPFC, dlPFC, dACC, TPJ, PCC, STRv

A, audio; I, image; T, text; C, Clip; P, full piece; S, still; ACC, anterior cingulate cortex; AMY, amygdala; dACC, dorsal anterior cingulate cortex; dlPFC, dorsolateral prefrontal cortex; DMN, default mode network; dmPFC, dorsomedial prefrontal cortex; EMN, empathy-related networks; FuG, fusiform gyrus; HPC, hippocampus; IFG, inferior frontal gyrus; ITC, inferotemporal cortex; MDN, medial dorsal nucleus; mPFC, medial prefrontal cortex; OFA, occipital face area; PC, parietal cortex; PCC, posterior cingulate cortex; PCun, precuneus; PMC, posterior medial cortex; STRv, ventral striatum; TL, temporal lobes; TPJ, temporoparietal junction.

**Table 2:** Risk of bias evaluation.

Study	Selection bias (sample size & diversity)	Performance bias (methodological rigor^a^)	Overall risk of bias
Altmann et al. (2012)	Moderate-small sample (24 participants in MRI)	Low-standard methods	Low to moderate
Chang et al. (2021)	Moderate-sample size reasonable (25 adults)	Low-behavioral + neuroimaging approach enhances validity	Moderate
Hartung et al. (2017)	Low-moderate sample (52 participants)	Low-well-established procedures	Low
Kaplan et al. (2017)	Low-large, cross-cultural sample (*N* = 78)	Low-use of real-life narratives enhances ecological validity	Low
Ohad and Yeshurun (2023)	Moderate-sample size reasonable (*N* = 25)	Low-behavioral + neuroimaging approach enhances validity	Moderate
Richardson et al. (2020)	Moderate-sample size reasonable (*N* = 37)	Low-behavioral + neuroimaging approach enhances validity	Moderate
Ron et al. (2022)	High-small sample (*N* = 17, 7 females), potential gender imbalance	Moderate-for role differentiation well-implemented	Moderate to high
Ryu and Kim (2024)	Moderate-use of two independent datasets (*N*1 = 16 & *N*2 = 23), but small size	Moderate-inter-subject correlation a strong approach, but sensitive to stimulus variability	Moderate
Vemuri and Surampudi (2015)	High-smallest sample (*N* = 15)	Moderate-cognitive-affective processing well-studied	High
Wagner et al. (2016)	Moderate-sample size (34) is reasonable but lacks diversity details	Low-reverse correlation enhances methodological robustness	Low to moderate
Weber et al. (2024)	Moderate-all-female sample (28 participants) limits generalizability	Low-robust approach with moral judgment paradigms	Moderate

a Most of the methods under fMRI.

### Shared neural patterns across studies

Across the reviewed studies, several neural regions consistently emerged as central parts of the narrative process. The mPFC was highlighted in nine studies as crucial for processing moral evaluation, emotional engagement and narrative integration (Altmann et al., 2012; Kaplan et al., 2017; Wagner et al., 2016). This region was found to play a crucial role in interpreting characters intentions and ethical dimensions, especially in morally complex storytelling scenarios, as demonstrated in studies such as Altmann et al. (2012), Kaplan et al. (2017), and Wagner et al. (2016). Additionally, the DMN was similarly prominent, appearing in eight studies (e.g., Richardson et al., 2020; Ryu and Kim, 2024), and played a key role in processing characters intentions, empathy-driven engagement, and mentalizing, enabling audiences to connect with characters internal states and motivations.

Other brain regions exhibited selective activation based on specific narrative contrasts. For instance, the IFG showed distinct activation patterns when differentiating protagonists from antagonists, reflecting its role in emotional empathy and information integration (Ryu and Kim, 2024; Altmann et al., 2012). The temporoparietal junction (TPJ) was frequently associated with perspective-taking and social categorization, particularly in narratives with morally ambiguous characters (Ron et al., 2022). In contrast, the ACC demonstrated a heightened variability during scenarios involving morally ambiguous antagonists, potentially reflecting increased cognitive conflict of moral dissonance (Weber et al., 2024).

### Protagonist vs. antagonist differentiation

Studies examining protagonist vs. antagonist differentiations revealed clear contrast in neural activation. On the one hand, protagonists elicited stronger synchronization in empathy-related regions such as the mPFC and IFG suggesting more straightforward emotional engagement (Ron et al., 2022; Ryu and Kim, 2024), demonstrating that protagonists tend to align with audiences’ expectations, resulting in predictable neural responses associated with empathy and narrative coherence.

On the other hand, antagonists triggered great activation in moral reasoning regions, including the ACC, dACC and PCC (Weber et al., 2024). These findings suggest that antagonists evoke a combination response between cognitive conflict with moral evaluation. For characters perceived as morally ambiguous, empathy-related networks (EMN) such as the medial dorsal nucleus (MDN) were activated, reflecting the audience’s attempt to reconcile conflicting moral cues (Vemuri and Surampudi, 2015). This aligns with Kaplan et al. (2017) studies, where TPJ activation was tied to the processing of morally complex narratives, highlighting the influence of narrative context on neural engagement.

### Functional differences based on narrative context

The format and context of the narrative stimuli were significantly influenced by the observed neural responses. Studies employing naturalistic stimuli, such as audiobooks (e.g., Ohad and Yeshurun, 2023) or dynamic film clips (e.g., Vemuri and Surampudi, 2015), demonstrated broader activation across networks like the DMN and TPJ. These multimodal stimuli enriched narrative engagement by replicating real-world storytelling, enabling participants to process characters intentions, social roles, and emotional dynamics mora effectively. Findings from Chang et al. (2021) and Ohad and Yeshurun (2023) emphasize the added depth and complexity afforded by naturalistic formats.

In contrast, controlled stimuli, including static images or text-based tasks, offered greater precision in isolating specific neural responses but engaged fewer brain regions. For example, Ron et al. (2022) and Kaplan et al. (2017) used more restrictive formats to focus on contrasts, such as moral vs. neutral values or protagonist vs. antagonists’ roles. This trade-off between the ecological validity and experimental control underscores the complexity of studying narrative engagement within distinct methodological frameworks.

Beyond stimulus format, narrative comprehension itself actively recruits a wide array of brain areas, including temporal and parietal regions for processing story content (Hartung et al., 2017) and affective networks for cognitive-affective integration (Vemuri and Surampudi, 2015). These studies collectively suggest that narrative engagement is not merely a passive experience but rather an active cognitive and affective process, recruiting distinct neural networks based on story elements, character roles, and moral context.

## Discussion

### Neural mechanisms of narrative engagement

The studies included in this systematic review reveal consistent neural patterns underlying character perception in narratives, with a particular emphasis on protagonist-antagonist differentiation. Across the reviewed studies, the DMN consistently emerges as a critical region in processing character engagement, social cognition, and moral reasoning (Ron et al., 2022; Richardson et al., 2020). Similarly, the mPFC plays a central role in evaluating the moral and emotional dimensions of characters, with distinct activations based on perceived likeability, empathy and ethical judgments (Kaplan et al., 2017; Altmann et al., 2012).

A notable observation pertains to the ACC, which exhibited increased variability in its response to antagonists compared to protagonists (Ron et al., 2022; Weber et al., 2024). This variability may suggest heightened cognitive conflict or moral dissonance. While antagonists often display morally ambiguous behaviors, the variability in the ACC could also depend on narrative context and how audiences perceive moral ambiguity, rather than being solely driven by character traits. This suggests a dynamic interplay between narrative cues and neural processing, which future studies should investigate through mediation analyses or correlational designs neuroimaging and behavioral measures.

Empathy also emerged as a significant factor in character engagement, with emotional empathy appearing more relevant than cognitive empathy for audience responses (Kaplan et al., 2017; Richardson et al., 2020). This finding aligns with prior literature suggesting that empathy-driven engagement is modulated by contextual and emotional resonance (Weber et al., 2024). In such manner, future investigations into how empathy subcomponents contribute to character perception and moral evaluation will be useful to advance the understanding of narrative engagement.

### Methodological challenges

The reviewed studies present several methodological limitations that constrain the generalizability and interpretability of the findings. Sample size limitations remain a significant concern, with several studies relying on small participation groups (e.g., Ron et al., 2022; Vemuri and Surampudi, 2015) to larger samples (e.g., Kaplan et al., 2017; Hartung et al., 2017). While larger samples enhance statistical power, the lack of power analyses in smaller studies raises questions about whether observed effects are adequately detected.

Participant diversity is another concern. For instance, Weber et al. (2024) exclusively examined female participants, potentially introducing selection bias and limiting generalizability to missed-gender populations. Similarly, Ron et al. (2022) included a gender-imbalanced sample, potentially skewing findings related to social cognition. Transparent reporting sample characteristics and statistically rigorous designs are essential for future research to improve representativeness.

Another methodological constraint is the variation in experimental paradigms. Studies using controlled stimuli, such as static images or text-based narratives, allow for precise variable isolation but they may not fully capture the complexity of real-world storytelling experiences (van Atteveldt et al., 2018; Nastase et al., 2020). Conversely, naturalistic stimuli like audiobooks and dynamic film clips provide richer emotional and contextual cues, but often lack experimental control (Ohad and Yeshurun, 2023; Chang et al., 2021). Balancing ecological validity and methodological rigor remains essential.

Specific paradigm examples illustrate these challenges. For instance, Ron et al. (2022) relied on static character images extracted from films, where participants viewed isolated faces of protagonists and antagonist while undergoing fMRI scanning. This approach, while allowing for controlled comparison of character types, neglects the influence of continuous action perception, dynamic facial expressions, and broader narrative context, factor that are crucial in real-world storytelling.

Other studies used video-based stimuli to examine neural responses to antagonists. Ryu and Kim (2024) presented participants with short film clips featuring social interactions between protagonist and antagonists, allowing for more dynamic character perception. This paradigm provided richer visual and emotional cues, engaging regions such as the IFG and ACC, however, the short duration of the clips may not fully capture long-term engagement with the characters in extended narratives.

Similar, Weber et al. (2024) focused on moral judgment by presenting participants with scenes depicting antagonists receiving punishment for immoral actions. This study provided insight into how audiences process justice-related events, activation brain regions associated with moral reasoning and vicarious justice. However, the reliance on specific moral scenarios may limit the applicability of findings to broader character perception processes.

Finally, Kaplan et al. (2017) used written narratives extracted from weblogs, where participants read short moral dilemmas involving different values. While this text-based approach allowed for precise control over linguistic content and cognitive processing, it needed multimodal engagement, potentially decreasing its ability to induce realistic social and emotional responses.

Prior research has suggested that employing more ecologically valid stimuli, such as full-length films and audiobooks, enhances the generalizability of fMRI findings beyond the artificial constraints of static visual inputs (van Atteveldt et al., 2018). While Ohad and Yeshurun (2023) adopted a more naturalistic approach by using an audio-based narrative derived from a previous study published by Chang et al. (2021), their study was still limited to a single storyline, restricting the broader applicability of their results. Additionally, one limitation of using fMRI is that it requires short, repeatable stimuli to obtain reliable signals. This makes it less suitable for studying longer, naturalistic narratives, where methods like EEG or fNIRS—better equipped to capture extended or continuous brain activity—are more appropriate.

## Conclusion and future directions

This systematic review synthesizes current neuroimaging research on narrative character perception, highlighting key contributions from the DMN, mPFC and ACC in differentiating protagonists and antagonists. These regions are central to empathy-drive engagement, moral reasoning and the dynamic interplay between audience and narrative context. Findings emphasize how antagonist evoke unique neural responses, engaging both cognitive and affective processes, and revealing the complexity of moral evaluation in narrative engagement.

This research offers insights into narrative processing that would be challenging to discern without fMRI. Specifically, it reveals distinct neural responses in the ACC and DMN that underscore the brain’s role in processing moral ambiguity, empathy and character differentiation. While it is intuitive that protagonists differ from antagonists, fMRI uncovers the nuanced interplay of neural networks—such as the variability of the ACC in morally ambiguous scenarios and the synchronization of the DMN during character engagement—driven by narrative complexity and moral reasoning. These findings illustrate how fMRI transcends behavioral observations, providing a deeper understanding of how neural mechanisms govern narrative engagement and character perception.

Future research should address methodological limitations by prioritize larger, and more diverse samples, transparent reporting practices, and longitudinal designs. Cross-cultural studies are specially promising for exploring how moral reasoning, narrative engagement and character perception evolves over time and across different cultural contexts. Additionally, immersive storytelling techniques, such as virtual reality (VR) or adaptive narratives, offer exciting opportunities to investigate narrative engagement and character perception in dynamic and ecologically valid settings.
